# Impact of two oral doses of 100,000 IU of vitamin D_3_ in preschoolers with viral-induced asthma: a pilot randomised controlled trial

**DOI:** 10.1186/s13063-019-3184-z

**Published:** 2019-02-18

**Authors:** Francine Monique Ducharme, Megan Jensen, Geneviève Mailhot, Nathalie Alos, John White, Elizabeth Rousseau, Sze Man Tse, Ali Khamessan, Benjamin Vinet

**Affiliations:** 1 Clinical Research and Knowledge Transfer Unit on Childhood Asthma, Research Centre, Sainte-Justine University Health Centre, Montreal, Quebec Canada; 2 Department of Pediatrics, University of Montreal, Sainte-Justine University Health Centre, 3175 Côte Ste-Catherine, Montreal, Quebec H3T 1C5 Canada; 3 Department of Social and Preventive Medicine, University of Montreal, Montreal, Quebec Canada; 4 Department of Nutrition, University of Montreal, Montreal, Quebec Canada; 5 Department of Physiology, McGill University, Montreal, Quebec Canada; 6 Euro-Pharm International Canada, Montreal, Quebec Canada

**Keywords:** Asthma, Child, Vitamin D, Cholecalciferol, Paediatric, Randomised controlled trial, Viral-induced, Pilot study

## Abstract

**Background:**

New evidence supports the use of supplemental vitamin D in the prevention of exacerbation of asthma; however, the optimal posology to sufficiently raise serum levels while maximising adherence is unclear. The objective was to ascertain the efficacy of high-dose vitamin D_3_ in increasing serum vitamin D in preschoolers with asthma and provide preliminary data on safety and efficacy outcomes.

**Methods:**

We conducted a 7-month, triple-blind, randomised, placebo-controlled, pilot trial of children aged 1–5 years with viral-induced asthma. Participants were allocated to receive two oral doses of 100,000 IU vitamin D_3_ (intervention) or identical placebo (control) 3.5 months apart, once in the fall and once in the winter. Serum 25-hydroxyvitamin D (25OHD) was measured by tandem mass spectrometry at baseline, 10 days, 3.5 months, 3.5 months + 10 days, and 7 months. The main outcome was the change in serum 25OHD from baseline (Δ25OHD) over time and at 3.5 and 7 months; other outcomes included the proportion of children with 25OHD ≥ 75 nmol/L, safety, and adverse event rates.

**Results:**

Children (*N* = 47) were randomised (intervention, 23; control, 24) in the fall. There was a significant adjusted group difference in the Δ25OHD (95% confidence interval) of 57.8 (47.3, 68.4) nmol/L, *p* < 0.0001), with a time (*p* < 0.0001) and group*time interaction effect (*p* < 0.0001), in favour of the intervention. A significant group difference in the Δ25OHD was observed 10 days after the first (119.3 [105.8, 132.9] nmol/L) and second (100.1 [85.7, 114.6] nmol/L) bolus; it did not reach statistical significance at 3.5 and 7 months. At 3.5 and 7 months, respectively, 63% and 56% of the intervention group were vitamin D sufficient (≥ 75 nmol/L) compared to 39% and 36% of the control group. Hypercalciuria, all without hypercalcaemia, was observed in 8.7% of intervention and 10.3% of control samples at any time point. Exacerbations requiring rescue oral corticosteroids, which appear as a promising primary outcome, occurred at a rate of 0.87/child.

**Conclusion:**

Two oral boluses of 100,000 IU vitamin D_3,_once in the fall and once in the winter, rapidly, safely, and significantly raises overall serum vitamin D metabolites. However, it is sufficient to maintain 25OHD ≥ 75 nmol/L throughout 7 months in only slightly more than half of participants.

**Trial registration:**

ClinicalTrials.gov, NCT02197702 (23 072014). Registered on 23 July 2014.

**Electronic supplementary material:**

The online version of this article (10.1186/s13063-019-3184-z) contains supplementary material, which is available to authorized users.

## Background

Asthma is the most common chronic childhood disease, affecting about 10% of children [1, 2], with preschoolers experiencing the highest rate of emergency department (ED) visits relative to other age groups [2–4]. Most exacerbations are triggered by viral upper respiratory tract infections (URTIs), particularly in young children [5, 6]. Circulating 25-hydroxyvitamin D (25OHD) has been inversely associated with an increased risk of viral URTIs, asthma severity, inhaled corticosteroid dose, and moderate or severe exacerbations [7–9], suggesting the potential role for vitamin D in viral infections and asthma. Inadequate dietary intake of vitamin D [10, 11], low use of vitamin D supplements [11, 12], dark skin pigmentation, obesity, and low sun exposure increase the risk of vitamin D insufficiency [13–15], affecting 40–82% of children living in high-latitude areas [11, 16, 17]. Of concern, is the higher reported rate of vitamin D insufficiency in children with versus without asthma [18–20]; in Canadian preschoolers with asthma, almost 75% are already vitamin D insufficient in the fall [21].

In a meta-analysis of individual patient data, Martineau and colleagues reported a significantly reduced risk of acute respiratory tract infections in participants receiving vitamin D supplementation [22]. A Cochrane systematic review of nine asthma trials (two adult and seven paediatric) [21], with variable use of inhaled corticosteroids (ICS), reported a statistically significant protective effect of vitamin D supplementation against exacerbations requiring rescue oral steroids or emergency department visits, with high-quality evidence and no heterogeneity [23]. However, the optimal posology of this promising strategy remains to be determined. We have demonstrated a rapid rise in serum vitamin D with the combination of a single bolus dose of 100,000 IU vitamin D_3_ and 400 IU daily for 6 months, but no significant group difference at 3 and 6 months compared to a placebo bolus and 400 IU vitamin D_3_ daily [21]. We surmised that the daily vitamin D supplementation in both groups attenuated group separation and that a single bolus was inadequate to maintain vitamin D sufficiency during both the fall and winter seasons.

The objective of this study was to determine if two bolus doses of vitamin D_3_ supplement_,_ once in the fall and once in the winter, are associated with a rapid and sustained improvement in serum 25OHD. Second, we wish to provide pilot data on the efficacy and safety of this intervention, before formally testing in an adequately powered trial the efficacy of this strategy to improve health outcomes in preschoolers with viral-induced asthma.

## Methods

### Design

We conducted a 7-month randomised, parallel-group, triple-blind, placebo-controlled trial at the Sainte-Justine University Health Centre (SJUHC), Montreal Canada, in accordance with Helsinki Good Clinical Practice Guidelines [24]. The Institutional Research Ethics Board (#2015–786, 4004) and Health Canada approved the study (#187438). Euro-Pharm (Montreal, Canada) donated the drug, but had no input into the study design, conduct, analysis, or writing of the study. Parents provided written informed consent for their child’s study participation and for the release of medical and pharmacy data. Parents received small monetary reimbursement for parking or transportation. The study is reported according to recommended standards (Additional file 1).

### Participants

Children aged 1–5 years were eligible if they had: (i) physician-diagnosed asthma, based on clinical signs of airflow obstruction and reversibility [25]; (ii) URTI reported by parents as the main asthma trigger; (iii) ≥ 4 URTIs in the preceding year; and (iv) ≥ 1 exacerbation requiring rescue oral corticosteroids (OCS) in the preceding 6 months (or ≥ 2 in the past 12 months), confirmed by pharmacy and/or medical records. Patients were excluded due to intake of or intention to use > 400 IU/day of vitamin D supplement; extreme prematurity (< 28 weeks’ gestation); high risk of vitamin D deficiency (e.g., vegan diet); condition(s) (e.g., rickets) or drug(s) altering calcium or vitamin D absorption or metabolism (e.g., anti-epileptic, diuretic, antacid, or anti-fungal medications); anticipated difficult follow up.

#### Randomisation and blinding

We randomised children to receive vitamin D or placebo supplement in a 1:1 ratio, using computer-generated random numbers with variable permuted blocks. No daily supplement was provided or recommended. The active (50,000 IU/mL of cholecalciferol) and placebo preparations were identical in appearance and taste. The Central Pharmacy (SJUHC) held the allocation codes, prepared the study supplements in sequentially coded syringes, and dispensed as per randomisation 2 mL of vitamin D_3_ (100,000 IU of cholecalciferol) or identical placebo, administered by the nurse at baseline and 3.5 months. At the end of follow up, parents, nurse, and physician independently guessed the child’s group assignment.

### Protocol

Children were randomised between 1 September and 30 November in 2014 and 2015 and were followed for 7 ± 0.5 months. Baseline characteristics included demographics, atopy [26], and recent morbidity. As per national recommendations physicians reviewed the management plan at randomisation to include daily ICS with/without adjunct therapy, or episodic high-dose ICS, with rescue salbutamol during exacerbations, delivered by metered dose inhalers with a holding chamber [25]. After randomisation, two medical visits at 3.5 ± 0.5 and 7 ± 0.5 months, with monthly phone contacts, served to review asthma control, vitamin D and calcium intake according to a validated food frequency questionnaire [27], URTIs, exacerbations, healthcare utilisation, and adverse health events. Non-fasting urine and blood samples were obtained at each visit (Additional file 2). At randomisation, the urine calcium:creatinine ratio (Ca:Cr) and serum calcium, phosphorus and alkaline phosphatase were systematically analysed. At each subsequent clinic and home visit, only urinary Ca:Cr was routinely analysed for safety monitoring; if abnormal, the aforementioned serum markers were analysed. Additional serum aliquots were stored at − 80 °C for total 25OHD vitamin D, which was analysed using tandem mass spectrometry only at the end of the study [28, 29].

### Outcomes

After premature trial cessation due partial funding enabling only a 2-year single-centre pilot trial, rather than an adequately powered multicentre study of 865 children, the primary outcome was modified post hoc to the overall change (∆) from baseline in total serum 25OHD and at 3.5 and 7 months, similar to our previous pilot study [21]. Post hoc secondary outcomes included group difference in the proportion of children with total 25OHD ≥ 75 nmol/L at 3.5 and 7 months, and the rate of OCS courses per child. Other outcomes specified a priori included the proportion of children with hypercalciuria (Ca:Cr) > 1.25 (1–2 years)or > 1 (2–5 years) nmol/nmol at any point in time; proportion of children with ≥ 1 exacerbation requiring rescue OCS (former primary outcome); number of emergency department (ED) visits; intensity and duration of asthma symptoms and cumulative use of rescue ß2-agonist use, documented on the *Asthma Flare-up Diary for Young Children* (ADYC) [30]; parental functional status during exacerbations ascertained on the *Effect of a child’s asthma flare-up on parents (ECAP)* [31]; and URTI duration.

### Statistical methods

An intention-to-treat (ITT) analysis was carried out whereby all randomised children were included in the analysis, wherever possible. The group difference in within-patient ∆25OHD level overall, and specifically at 3.5 and at 7 months, was examined using a generalised linear mixed model, after adjustment for variables with a potential for effect modification (vitamin D intake, ethnicity) or baseline group imbalance (sex, ethnicity, environmental tobacco exposure, school-day missed, asthma management strategy). Modified multivariable logistic regression (to deal with lack of convergence) [32] served to estimate the relative risk of children experiencing at least one event (OCS and emergency department (ED) visits), with 95% confidence interval. We computed the incidence rate ratio to compare the event rate per child, namely, the mean number of OCS and URTI occurring during the 7-month follow up, with an offset variable for variations in person-time, where relevant. The severity and duration of asthma symptoms, use of rescue β_2_-agonists, and functional outcomes during episodes were compared across groups using a generalised linear regression model, adjusting for the clustering of events in individual children. In all efficacy models, covariates considered a priori for inclusion in the model were those with a potential for effect modification: vitamin D intake, asthma phenotype (episodic versus persistent), skin colour (Fitzpatrick scale) [33], or baseline group imbalance (sex, ethnicity, environmental tobacco exposure, school-day missed, management strategy (episodic ICS versus daily ICS monotherapy versus daily ICS with adjunct therapy)). Of note, to avoid multicollinearity due to the strong correlation (*r* = 0.95) between ethnicity and skin colour, ethnicity was selected, because complete data were available. Continuous values were displayed as mean (95% CI). All tests were two-sided with estimates presented with 95% CI, with no adjustment for multiple outcomes. All analyses were carried out using SAS software version 9.3.

## Results

**Fig. 1:**
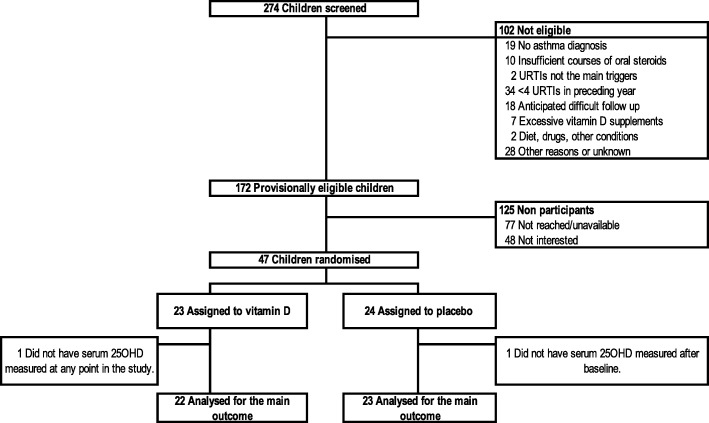
Patient selection. The flow of patients is depicted from screening to analysis; 274 children were screened, 102 were not eligible (non-mutually exclusive reasons for ineligibility are listed). Of the 172 provisionally eligible children, 77 could not be reached to confirm eligibility and 48 were not interested in study participation. Of the 47 randomised children, 23 were allocated to receive vitamin D and 24 to receive placebo supplementation. With one child in each group with no measurement of serum 25-hydroxyvitamin D (25OHD) after baseline, 22 and 23 children in the vitamin D and placebo groups were analysed for the main outcome. URTI, Upper respiratory tract infection

We screened 274 children: 102 were ineligible, primarily due to an insufficient number of URTI (33%), no asthma diagnosis (20%), anticipated difficult follow up (18%), and no recent rescue OCS use (10%). Of the 172 potentially eligible children, 125 declined participation mainly because of the number of blood tests, lack of time to comply to other procedures, and lack of interest. Participants were comparable to non-participants in age and sex (data not shown). There were 47 children randomised to the intervention (*N* = 23) or control group (*N* = 24) (Fig. 1).

Most baseline characteristics were similar between groups but some appeared slightly imbalanced, with a greater proportion of male participants, environmental tobacco exposure, use of combination therapy, more school days missed, fewer Caucasians and lower vitamin D dietary intake in the intervention compared to the placebo group (Table 1). With a mean age of 2.9 years, most participants were male, Caucasian, and had persistent asthma and URTI as one of multiple asthma triggers; half were atopic. Participants had experienced significant morbidity in the preceding year and at randomisation, most were prescribed daily ICS with or without adjunct therapy. Barely 13% were taking supplemental vitamin D at baseline at a daily dose of 50–400 UI. The median dietary intake of vitamin D (< 250 IU) was markedly below the recommended 600 IU [34] and did not change significantly throughout the study. Most participants had vitamin D insufficiency (< 75 nmol/L) at randomisation, but none had deficiency (< 25 nmol/L) [35].

**Table 1:** Baseline subject characteristics

	Vitamin D	Placebo
	(*N* = 23)	(*N* = 24)
Demographics		
Age (years) - mean ± SD	2.9 ± 0.9	2.9 ± 1.2
Male gender - *n* (%)	16 (70)	14 (58)
Caucasian ethnicity - *n* (%)	12 (52)	16 (67)
Skin colour^a^ − *n* (%)	*N = 22*	*N = 22*
1–2	12 (55%)	15 (68%)
3–6	10 (45%)	7 (32%)
Family history of asthma - *n* (%)	7 (30)	10 (42)
Environmental smoke exposure^b^ − *n* (%)	9 (39)	6 (25)
Daycare attendance - *n* (%)	20 (87)	22 (92)
Asthma morbidity in previous 12 months - median number (25%, 75%)		
Emergency department visits	4 (3, 5)	4 (2.5, 5.5)
Courses of oral corticosteroids	2 (1, 3)	2 (1, 3)
Hospital admissions	1 (0, 2)	0 (0, 1)
School or day-care days missed	10 (4, 15)	6 (3, 10)
Assessment at randomisation		
Persistent symptoms - *n* (%)	16 (73)	17 (71)
Multi-trigger asthma - *n* (%)	13 (57)	13 (54)
Atopy^c^ - *n* (%)	12 (52)	12 (50)
Influenza immunisation - *n* (%)	3 (13)	3 (13)
Prescribed asthma controller - *n* (%)^d^		
Episodic ICS	2 (9)	2 (8)
Daily ICS monotherapy	15 (65)	20 (83)
Daily ICS combination therapy^e^	6 (26)	2 (8)
Dietary status		
Vitamin D intake, IU/day - median (25%, 75%)	182 (125, 425)	238 (162, 270)
Serum vitamin D < 75 nmol/L - *n* (%)	15 (68)	13 (54)

*ICS* inhaled corticosteroids

^a^Ascertained by the 6-point Fitzpatrick’s sun-reactive skin type classification from (1) very light skin to (6) dark skin [33]

^b^Environmental smoke exposure *in utero* or currently in house or car

^c^Defined as reported hay fever or eczema on the International study of asthma and allergies in childhood (ISAAC) questionnaire [26], environmental or food allergy, blood eosinophils counts > 0.4/uL

^d^Two patients in the intervention group refused recommended treatment (one prescribed episodic ICS was allowed to take no medication and one prescribed daily ICS was allowed to take daily montelukast, both until reassessment or the next exacerbation); they were classified in their respective recommended therapy

^e^ICS in combination with long-acting β2-agonist or montelukast

The median (25%, 75%) follow up was 7 (6.5, 7.4) months, with 89% retention: one participant from the intervention group withdrew at visit 1, two participants (one each from the intervention and placebo group[s) withdrew at visit 2, before the first and second bolus, respectively (due to fear/inconvenience of blood tests), and two participants from the intervention group withdrew before visit 3 (due to the inconvenience of medical visits). After excluding doses not given due to study withdrawals, full-bolus retention was documented in 94% (85/90) of administered boluses: four patients (two each from the intervention and placebo groups) partially spit five doses. There was no evidence of unblinding among participants, nurses, or physicians (Additional file 3).

**Fig. 2:**
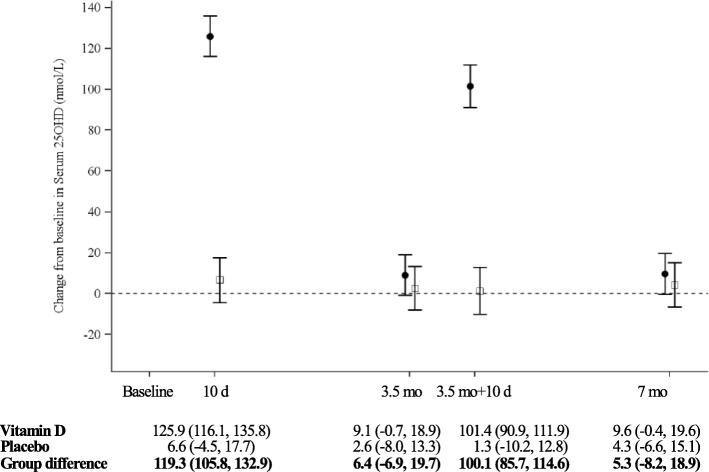
Change from baseline in serum 25-hydroxyvitamin D (25OHD) levels over 7 months. The adjusted mean change from baseline in 25OHD is presented with 95% confidence interval at each time point, in the vitamin D (filled circles) and placebo (open squares) groups over the 7-month study period; values were adjusted for vitamin D intake, ethnicity, sex, environmental tobacco exposure, school-days missed, and asthma management strategy. In the lower panel, the adjusted mean changes from baseline (95% CI) for each group and the adjusted mean group difference in the change from baseline (95% CI) are recorded at 10 days (d) (after 1st bolus), 3.5 months (mo), 3.5 months + 10 days (after 2nd bolus) and 7 months

**Fig. 3:**
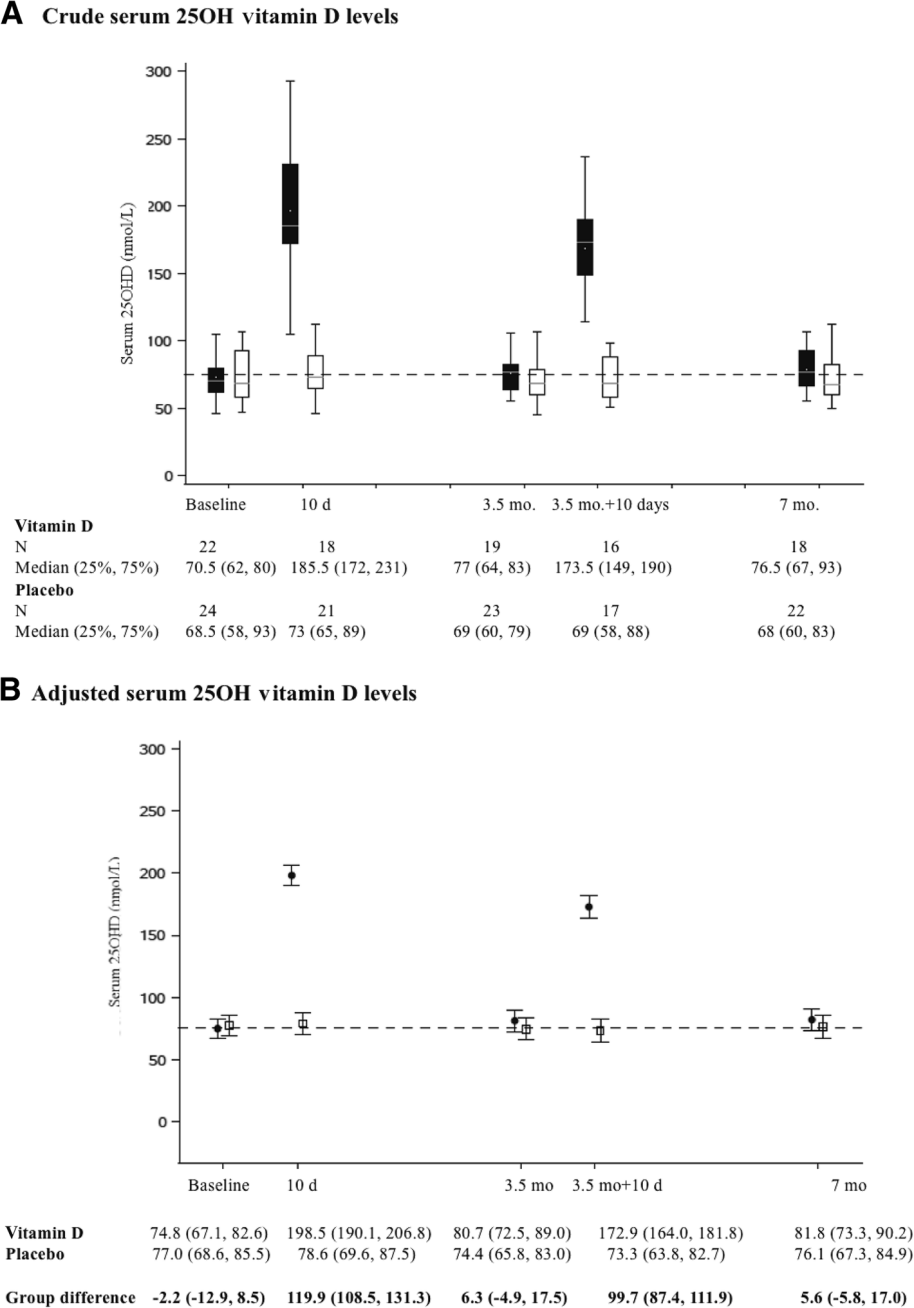
Serum 25-hydroxyvitamin D (25OHD) levels over 7 months. The 25OHD values are presented by group at various time points. **a** Crude total serum 25OHD in the vitamin D (filled boxes) and control (open boxes) groups over the 7-month study period. The median is depicted by the horizontal bar, with the lower and upper limits of each box representing the 25% and 75%; these numerical values are also recorded in the lower panel as median (25%, 75%) at each time point. Error bars represent the minimum and maximum of the distribution. **b** Adjusted marginal means for the total serum 25OHD are presented in the vitamin D (filled circles) and control (open squares) groups over the 7-month study period, after adjustment for asthma phenotype, sex, ethnicity, environmental tobacco exposure, school-days missed, baseline vitamin D intake, baseline serum 25OHD, and asthma management strategy. Error bars represent the 95% confidence interval of the mean. In the lower panel, their numerical values are recorded by group at each point in time along with the adjusted mean group difference (95% CI). Using a generalised linear mixed model, a statistically significant overall group (*p* < 0.0001), time (*p* < 0.0001), and group*time (*p* < 0.0001) interaction was documented. In both graphs, the dotted line represents the 75 nmol/ L on the y-axis. Total 25OHD was comprised overwhelmingly of 25-hydroxyvitamin D_3_, with 3-epimer-25-hydroxyvitamin D_3_ and 25-hydroxyvitamin D_2_

There was an overall statistically significant group difference in total serum ∆25OHD over time (57.8 (47.3, 68.2) nmol/L, *p* < 0.0001), with a significant time (*p* < 0.0001) and group*time interaction (*p* < 0.0001) effect (Fig. 2). Ten days following the first and second bolus, the group difference in the adjusted within-patient mean ∆25OHD from baseline was 119.3 (95% CI 105.8, 132.9) nmol/L and 100.1 (95% CI 85.7, 114.6) nmol/L, respectively, whereas the mean ∆25OHD at 3.5 months (6.4 (− 6.9, 19.7) nmol/L) and 7 months (5.3 (− 8.2, 18.9) nmol/L) was not statistically significant. The same held true when comparing crude or adjusted 25OHD serum values at various time points between groups (Fig. 3). All patients in the intervention group became vitamin D sufficient (≥ 75 nmol/L) 10 days after the first and second bolus compared to 48% (*p* = 0.0003) and 35% (*p* < 0.0001) of patients in the control group, respectively. Vitamin D sufficiency was maintained at 3.5 months in 63% vs. 39% (*p* = 0.12) and at 7 months in 56% vs. 36% (*p* = 0.22) in the intervention versus the control groups, respectively.

Over the 7-month study period, patients experienced an average of 4 URTIs and 2–3 viral-induced asthma exacerbations, with no significant group difference in incidence. URTIs were more frequently reported verbally during monthly contact than documented on diaries. Participants experienced 41 exacerbations requiring rescue OCS (mean: 0.87/patient); more than half of participants made an ED visit and received OCS for asthma. No significant group difference was observed in healthcare utilisation, severity and average duration of asthma episodes, use of β2-agonists, or lost workdays or functional status of caregivers; observations were consistent across data sources (verbal report versus diaries) (Table 2).

**Table 2:** Efficacy outcomes

	Vitamin D		Placebo		Adjusted incidence rate ratio	Adjusted relative risk	Adjusted mean difference
	events (number)	Mean (95% CI) *N* = 23	events (number)	Mean (95% CI) *N* = 24	(95% CI)	(95% CI)	(95% CI)
URTI episodes^a^							
Incidence of URTIs/child^b^							
Verbal report - mean group rate/child	98	4.48 (3.66, 5.49)	95	4.12 (3.37, 5.03)	1.24 (0.88, 1.75)		
Diary - mean group rate/child^c^	44	3.63 (2.70, 4.87)	64	4.24 (3.32, 5.42)	0.92 (0.58, 1.49)		
Asthma exacerbations with URTIs^a^							
Incidence of exacerbations/child^b^							
Verbal report - mean group rate/child	61	2.84 (2.20, 3.67)	44	1.91 (1.42, 2.56)	1.78 (1.10, 2.90)		
Diary - mean group rate/child^c^	33	2.72 (1.93, 3.83)	52	3.71 (2.83, 4.87)	0.74 (0.43, 1.27)		
Intensity of asthma symptoms/episode^d^							
Diary - cumulative daily ADYC scores^e, c^	34	11.0 (8.9, 13.2)	54	12.5 (9.8, 15.1)			−0.7 (−3.7, 2.2)
Duration of exacerbations in days^d^							
Verbal report - mean group duration/episode	57	7.6 (5.1, 10.0)	43	7.4 (5.8, 9.0)			1.0 (−3.0, 4.9)
Diary - mean group duration/episode^d, c^	34	5.1 (3.1, 7.0)	54	5.2 (4.2, 6.2)			0.3 (−1.9, 2.6)
Intensity of *β*2-agonist use per episode^f^							
Diary - cumulative number of puffs^c^	33	40.1 (30.4, 49.8)	51	41.2 (32.1, 50.2)			−1.4 (− 13.5, 10.7)
Impact on parents during URTI^a^							
Parental functional status^g^	35	3.3 (2.8, 3.9)	49	3.2 (2.6, 3.8)			−0.1 (−1.3, 1.0)
Workday-missed/episode	36	1.0 (0.3, 1.7)	50	1.7 (0.9, 2.5)			0.1 (−1.2, 1.3)
Healthcare service utilisation							
Courses of oral steroids/child	22	0.96 (0.63, 1.45)	19	0.79 (0.51, 1.24)	1.21 (0.57, 2.57)		
Acute care visits for asthma/child	24	1.04 (0.70, 1.56)	24	1.00 (0.67, 1.49)	1.34 (0.69, 2.63)		
		Proportion (95% CI)		Proportion (95% CI)			
Subjects with ≥ 1 course of oral steroids	14	61 (40, 78) %	10	42 (24, 62) %		1.36 (0.42, 4.47)	
Subjects with ≥ 1 acute care visit	15	65 (44, 82) %	12	50 (31, 69) %		1.63 (0.51, 5.21)	

Values for each group are reported as mean (95% CI). Unless otherwise specified, all summary estimates (odds ratio, rate ratio, and mean difference) were analyzed by intention-to-treat with adjustment for the clustering of upper respiratory tract infections (URTIs) by individuals and offset to account for varying person-time, when applicable

*ADYC* Asthma flare-up diary for young children [30]

^a^An upper respiratory tract infection (URTI) was deemed to have occurred when reported by parents at monthly contacts or in diaries. The number of URTIs for which a complete set of diaries was available is indicated for each outcome

^b^Standardised over 210 days (i.e., 7 months, the expected duration of the study) to account for varying person-time

^c^Children who did not complete any asthma diaries were excluded from the calculation of the summary estimates

^d^Duration from the first day with two or more asthma symptoms to the last day with one or more asthma symptoms (cough, wheezing, and/or dyspnea) as reported by parents on the monthly contact and/or diary. Up to one day without asthma symptoms could be included in an exacerbation. URTIs with incomplete reports and diaries were discarded

^e^Measured on the 17-item ADYC [30], on a scale of 1 (best) to 7 (worst), completed daily from the beginning until the end of asthma symptoms during exacerbation. The cumulative symptoms intensity represents the sum of the daily ADYC scores per episode (URTI or exacerbation). The ADYC items pertained to the cough (*N* = 2), wheezing (*N* = 2), dyspnea (*N* = 4), night awakenings (*N* = 1), general wellbeing (*N* = 5), and child’s response to albuterol inhalations (*N* = 3)

^f^Cumulative number of inhalations during asthma exacerbations, standardized over 7 days, as recorded on the ADYCs. Albuterol doses received during acute care visits and hospital admissions were not considered. URTIs with missing or incomplete ADYCs were discarded

^g^Average score on 'Effects of a Young Child’s Asthma Flare-up on the Parents, (ECAP) Questionnaire (best = 7, worst =1)

Urinary Ca:Cr was normal in all but 9/104 (8.7%) intervention samples (*N* = 4 patients) and 12/117 (10.3%) placebo samples (*N* = 8 patients) at baseline or after randomisation, all with borderline abnormal values; no episode of hypercalciuria was associated with hypercalcaemia, decrease in alkaline phosphatase, or elevated (> 225 nmol/L) 25OHD, with one exception where the 25OHD was 231 nmol/L. Six patients in the intervention group had a 25OHD value > 225 nmol/L 10 days after the initial (*N* = 5 patients: 231–293 nmol/L) or the second (*N* = 1 patient: 237 nmol/L) bolus; only one episode was associated with borderline hypercalciuria (Ca:Cr 1.02) and none was associated with hypercalcaemia. Overall, 227 adverse health events were reported using MedDRA [36] (intervention, 96; control, 131), the most frequent being infections (*N* = 88, 39%) and general disorders (*N* = 47, 21%) (Additional file 4); one serious adverse health event, hospitalisation for pneumonia, occurred in the placebo group.

## Discussion

In this group of high-morbidity preschool children with recurrent viral-induced exacerbation of asthma, two boluses of 100,000 IU vitamin D_3_ given once in the fall and once in the winter rapidly raised serum 25OHD and increased overall 25OHD compared to a placebo. However, at 3.5 months and 7 months the residual change from baseline in serum 25OHD was only modest and in both cases was not significantly different from that in the placebo group. This supplemental approach permitted the majority of, but not all, patients in the intervention group to achieve and maintain vitamin D above 75 nmol/L throughout the study.

Children living in countries at high latitude are recommended to take vitamin D supplementation during the fall and winter to maintain serum 25OHD at or above 75 nmol/L for bone health [35]. Despite the recommended average dietary requirement of 600 IU in children ≥ 1 year of age as set by the Institute of Medicine [37], our participants’ dietary intake was less than half the recommended intake, with barely 13% taking a vitamin D supplement, a finding concordant with that of our prior pilot study [21]. The majority of children were already vitamin D insufficient at baseline in the fall, probably as a result of a combination of factors, including low intake, sun protection practices, and skin colour [11, 38]. The lower dietary intake and lower serum 25OHD observed in participants contrasts with that of healthy and population-based Canadian preschoolers [39]. Perhaps the avoidance of milk due to the widespread belief that dairy products increase mucus production [40, 41] or less time spent outdoors because of respiratory symptoms triggered by physical activities or environmental allergies contribute to these findings in our young population in whom half were atopic. Nonetheless, our results indicate that preschoolers with asthma are at high risk of vitamin D insufficiency.

A bolus dose of 100,000 IU vitamin D, once in the fall and once in the winter, is recommended by the French Society of Paediatrics for healthy preschoolers [42] and has been shown to rapidly and safely raise blood 25OHD levels. With recent meta-analyses suggesting a beneficial effect of supplemental vitamin D on reducing the incidence of viral infections [22] and asthma exacerbations [23], a rapid increase in circulating 25OHD may be ideal to efficiently prevent the well-documented paediatric “September epidemic” of viral-induced asthma exacerbations in the Northern hemisphere [43]. Although the optimal posology to prevent asthma exacerbations is unknown, bolus doses previously appeared less protective against respiratory infections than daily doses in children; however, this approach was primarily tested in undernourished children as a means to prevent pneumonia [44, 45]. In contrast, a 2015 meta-analysis of paediatric trials strongly recommended the use of a bolus dose (< 300,000 IU), concluding that daily doses as high as 4000 IU were insufficient to rapidly raise serum 25OHD in vitamin D deficient children [46]. The present trial confirms that each loading dose of 100,000 IU rapidly raises serum 25OHD in predominantly vitamin D insufficient children, but is inadequate to maintain sufficiency in about 40% of children; indeed, only a nominal change from baseline of approximatively 5–6 nmol/L was maintained 3.5 months after each bolus. Our findings of an acute rise in 25OHD following each bolus is concordant with the prior literature [21, 47], as is the return to near baseline levels approximately 3.5 months post bolus [48, 49]. Despite our hope to avoid the need for daily supplementation, two boluses proved suboptimal in many young asthmatic children.

The combination of bolus and daily supplementation would appear more promising. Indeed, in our prior pilot study of asthmatic preschoolers [21], the control group, receiving only 400 IU daily vitamin D (with no bolus), had a slow but steady increase from baseline of 20 nmol/L at 3 months, with no further improvement despite ongoing supplementation; yet, only 55% reached 75 nmol/L after 3 months of supplementation, confirming this approach as suboptimal. In contrast, the intervention group in the same trial, receiving a single 100,000 IU bolus combined with 400 IU daily vitamin D_3_, displayed a rapid rise of 132 nmol/L in serum 25OHD within 10 days, with a clinically meaningful change from baseline of 27.1 nmol/L at 3 months, but little additional increase to 6 months. Whereas all patients in the intervention group had maintained serum 25OHD at ≥ 75 nmol/L at 3 months, it dropped to 88% by 6 months, suggesting the need for a second bolus. Admittedly, the target serum level for the immune and anti-inflammatory effect observed with vitamin D supplementation by Martineau and colleagues [22, 23] remains to be established. Collectively, the findings of our two pilot studies would support two loading doses of 100,000 IU spaced 3.5 months apart, combined with a daily dose of 400 IU throughout the fall and winter, as an optimal intervention to achieve a rapid and sustained increase in serum vitamin D in preschoolers with asthma.

No significant group difference was observed in the severity of asthma exacerbations or healthcare utilisation. The non-significant trend observed in several outcomes, which was not in favour of vitamin D, is likely random, but may also be explained by baseline group imbalances (despite adjustment); more children in the intervention group displayed several baseline characteristics associated with more severe asthma and lower vitamin D exposure (skin colour and dietary intake) compared to the control group.

The study contributes to accumulating evidence on the safety profile of 100,000 IU vitamin D boluses. Although several participants displayed elevated urinary Ca:Cr, blood calcium levels were normal in all cases, suggesting that hypercalciuria was perhaps due to an unbalanced diet or a non-fasting state. Moreover, all participants with elevated serum 25OHD had normocalcaemia. To date, only five published paediatric trials have tested an oral dose of 100,000 IU vitamin D, with just two reporting circulating 25OHD: two cases (0.3%) with 25OHD > 375 nmol/L occurred in one trial of malnourished Afghan infants [44] and two children (18%) with 25OHD > 250 nmol/L but no associated hypercalciuria or hypercalcaemia were reported in our prior pilot study [21]. In the three small trials reporting serum bone metabolism biomarkers in the paediatric population, no episodes of hypercalcaemia occurred [47, 49, 50]. Yet, additional safety data in larger samples are required.

We acknowledge several trial limitations and lessons learned. Due to its small sample size, this study resulted in baseline group imbalances and provided only preliminary process and efficacy data. However, in line with our prior trials using similar criteria [21, 50], the high morbidity of enrolled patients, evidenced by the frequency of exacerbations requiring rescue OCS in the preceding 12 months (≥ 2/child) and during the study period (0.87/child), confirmed the appropriateness of eligibility criteria and the ongoing health burden, despite ICS therapy. With two thirds of participants already vitamin D insufficient at baseline and the expected further decline during the winter, the pragmatic recruitment without pre-screening for vitamin D status appears justified. Whereas greater efficacy had been suggested in asthmatic patients with the lowest baseline 25OHD [23], randomising children irrespective of baseline vitamin D would provide the opportunity to explore the dose-response association with, and identify the optimal target for, immune and anti-inflammatory effect. The absence of clinically important change in dietary vitamin D intake over the study period suggests no evidence of Hawthorne bias. Tolerance of the 2-mL bolus was excellent with 94% of administered doses completely retained. Importantly, we documented the main outcome in all participants, despite an 11% dropout, with pre-approved consent to obtain drug and medical information at study endpoint. The twofold higher number of URTI and asthma exacerbations documented by monthly verbal versus diary report, suggests the need to facilitate diary completion, perhaps by offering electronic diaries, shown to improve veracity and adherence [51, 52]. Conducted in a single centre with multi-ethnic Canadian preschoolers with asthma, most of whom were vitamin D insufficient at baseline, the observed serum 25OHD response to two boluses of 100,000 IU may not apply to healthy children, other ethnic groups, or those with significantly higher (or lower) baseline vitamin D status or exposure. These lessons learned have been implemented in the ongoing, funded, large, multicenter, triple-blind, placebo-controlled trial testing two boluses of 100,000 IU with daily 400 IU of vitamin D in the same population (NCT 03365687).

## Conclusion

The administration of an oral bolus of 100,000 IU vitamin-D_3_ in the fall, with a repeat dose in the winter, rapidly and significantly raises overall serum 25OHD in Canadian preschoolers with high-morbidity asthma, despite suboptimal dietary and sun exposure. While sufficient in slightly more than half of children, this strategy appears inadequate to maintain vitamin D sufficiency over 7 months in a notable proportion of this population.

## Additional files

Additional file 1: Consolidated Standards of Reporting Trials (CONSORT) 2010 checklist of information to include when reporting a randomised trial. (DOC 217 kb)

Additional file 2: Study intervention and procedures. (xlsx 14 kb)

Additional file 3: Study mechanics. (xlsx 15 kb)

Additional file 4: Adverse health events. (xlsx 13 kb)

## Abbreviations

25OHD: 25-Hydroxyvitamin D
ED: Emergency department
ICS: Inhaled corticosteroids
OCS: Oral corticosteroids
URTI: Upper respiratory tract infection
